# Periventricular leukomalacia-related litigation in Japan: a qualitative analysis of causation under clinical uncertainty

**DOI:** 10.3389/fped.2026.1907180

**Published:** 2026-08-12

**Authors:** Shigeo Iijima

**Affiliations:** Department of Regional Neonatal-Perinatal Medicine, Hamamatsu University School of Medicine, Hamamatsu, Japan

**Keywords:** causation, cerebral palsy, medical litigation, perinatal care, periventricular leukomalacia, preterm infant

## Abstract

**Introduction:**

Periventricular leukomalacia (PVL) is a leading cause of cerebral palsy in preterm infants. However, its multifactorial pathogenesis and uncertainty regarding the timing of brain injury make causal attribution in medical litigation inherently complex. Despite its clinical significance, little is known about how courts evaluate causation and responsibility in PVL-related cases. This study aimed to clarify the clinical and judicial characteristics of PVL-related medical litigations in Japan.

**Methods:**

Cases were identified using a nationwide legal database. A qualitative analysis of judicial decisions was performed, focusing on obstetric and neonatal management, breach of duty, causation, and informed consent. Seven litigation cases involving preterm infants were included.

**Results:**

Despite substantial heterogeneity in clinical presentation and perinatal course, cross-case analysis identified several recurring patterns in judicial reasoning regarding causation and responsibility. Disputed issues arose across multiple phases of perinatal care, including the timing of delivery, fetal monitoring, maternal transfer, postnatal care systems, and respiratory management. Although judicial decisions showed considerable variability, the recognition of causation emerged as the pivotal determinant of liability. In some cases, causation was acknowledged only partially, despite persistent clinical uncertainty regarding the timing and mechanisms of injury. In contrast, in cases involving clearly identifiable intrapartum hypoxic events, courts were more likely to affirm causation by linking clinical events in a coherent temporal sequence despite the underlying medical uncertainty.

**Discussion:**

These findings demonstrate that PVL-related litigation may involve disputes across multiple phases of perinatal care because of the multifactorial nature of PVL and the uncertainty regarding the timing and etiologies of injury. Under such conditions of clinical uncertainty, legal responsibility is assessed not only through isolated medical acts but also through broader clinical decision-making processes involving critical decisions across multiple phases of perinatal care. Moreover, the results highlight a fundamental divergence between medical reasoning based on scientific evidence and legal reasoning. The findings underscore the importance of transdisciplinary decision-making, careful documentation, and effective communication in managing medico-legal risk in perinatal practice.

## 1 Introduction

Periventricular leukomalacia (PVL) is a central nervous system injury that primarily affects infants born before 33 weeks of gestation and is characterized by ischemic lesions in the periventricular white matter. This condition is a major cause of cerebral palsy (CP), particularly spastic diplegia and spastic quadriplegia (1). Pathologically, PVL is characterized by coagulative necrosis, microglial infiltration, astrocyte proliferation, and cyst formation (2). Banker and Larroche first described PVL in 1962: they identified necrotic lesions in the periventricular white matter of premature infants and suggested its clinical association with CP (3). In the 1980s, advances in ultrasonography enabled bedside cranial ultrasound examinations, allowing PVL to be diagnosed relatively early in the postnatal period (4, 5). Furthermore, the introduction of magnetic resonance imaging in the late 1980s markedly improved diagnostic accuracy (6–8), which helped establish PVL as a major etiological factor for CP among preterm infants.

The pathogenesis of PVL is multifactorial, involving immature cerebral vascular architecture, vulnerability of oligodendrocytes, hypoxia–ischemia, and inflammation (9), among other factors (1, 10). The timing of injury varies widely, ranging from the fetal period to the postnatal stage. Although studies have attempted to elucidate the timing of insult (11, 12), precise determination remains difficult in many cases. In addition, the characteristic imaging findings of PVL are often not apparent until 2–3 weeks after birth, and many affected infants remain asymptomatic for several months before presenting with developmental delays or motor impairment (13). These sources of clinical uncertainty—including causation, the timing of injury, and long-term prognosis—together with potential differences in understanding between healthcare professionals and families, contribute to the emergence of medico-legal disputes.

Recent advances in neonatal care have improved the survival of preterm infants. However, this progress has been accompanied by an increase in medical disputes related to neurological sequelae, and numerous cases of litigation involving CP have been reported (14, 15). In PVL-related litigation, key issues include whether responsibility lies in obstetric management or postnatal care and how causation should be legally evaluated in conditions with substantial medical uncertainty. Understanding how the judiciary evaluates medical practice under conditions of clinical uncertainty is essential for improving the quality of perinatal care, supporting clinical decision-making, and enhancing medico-legal risk management. Nevertheless, systematic legal analyses specifically focusing on PVL remain limited. This study qualitatively analyzed PVL-related medical litigation cases in Japan, focusing on the assessment of causation and attribution of responsibility. The aim was to clarify how Japanese courts construct causation and attribute legal responsibility under conditions of clinical uncertainty and to explore the implications for perinatal practice.

## 2 Materials and methods

### 2.1 Data source and case identification

This study was designed as a qualitative analysis of publicly available judicial decisions related to PVL. Cases of PVL-related medical litigation were identified using D1-Law.com (Daiichi Hoki, Tokyo, Japan) (16), a comprehensive legal research engine widely used in Japan for studies on medical malpractice (17) that covers more than 360,000 judicial decisions, including cases dating back to the prewar period. A full-text keyword search of judicial decisions was performed using the single term “periventricular leukomalacia” to identify relevant medical malpractice cases. The search was performed without date restrictions. Only cases for which the full text of the judicial decision was accessible were included. Cases were included only when PVL had been diagnosed and explicitly described in the judicial decision. Because this study was based on judicial decisions rather than original clinical records, PVL diagnosis was accepted as described in the court documents and related medical records cited within the judgments. No independent review of neuroimaging findings, diagnostic validity, or maternal and obstetric histories was performed because original medical records were unavailable and the analysis relied exclusively on information documented in the judicial decisions. Preterm PVL cases were identified based on gestational age information described in the judicial decisions. Retrieved cases were excluded if (A) PVL was not diagnosed; (B) PVL was diagnosed but unrelated to the subject of litigation; or (C) litigation-related events occurred after the neonatal period (defined as >28 days after birth). Ultimately, only litigation cases involving preterm infants with PVL were included in this analysis. Where necessary, supplementary information was obtained from additional sources, including Hanrei Jiho, Hanrei Times, and Ichushi Web (Japan Medical Abstracts Society database), to complement details from judicial decisions.

As Japanese judicial decisions are indexed inconsistently and PVL-related litigation is relatively rare, a broad single-keyword search strategy was adopted to maximize sensitivity. Nevertheless, it is possible that some cases were missed if PVL was not explicitly described in judicial records.

### 2.2 Data extraction and analysis

For each included case, the following clinical and legal variables were extracted: birth year, gestational age, birth weight, clinical course, year filed, key issues in dispute, court decisions, awarded compensation, and, where available, maternal medical history, obstetric history, placental findings, and presence of pregnancy complications or fetal abnormalities. The qualitative analysis focused on the following predefined domains: (A) type of disputed issue (obstetric or neonatal management), (B) presence or absence of breach of duty of care, (C) evaluation of causal relationship, and (D) role and interpretation of the duty to provide explanation (informed consent). These elements were analyzed descriptively to identify patterns in judicial reasoning and medico-legal evaluation.

## 3 Results

**Figure 1:**
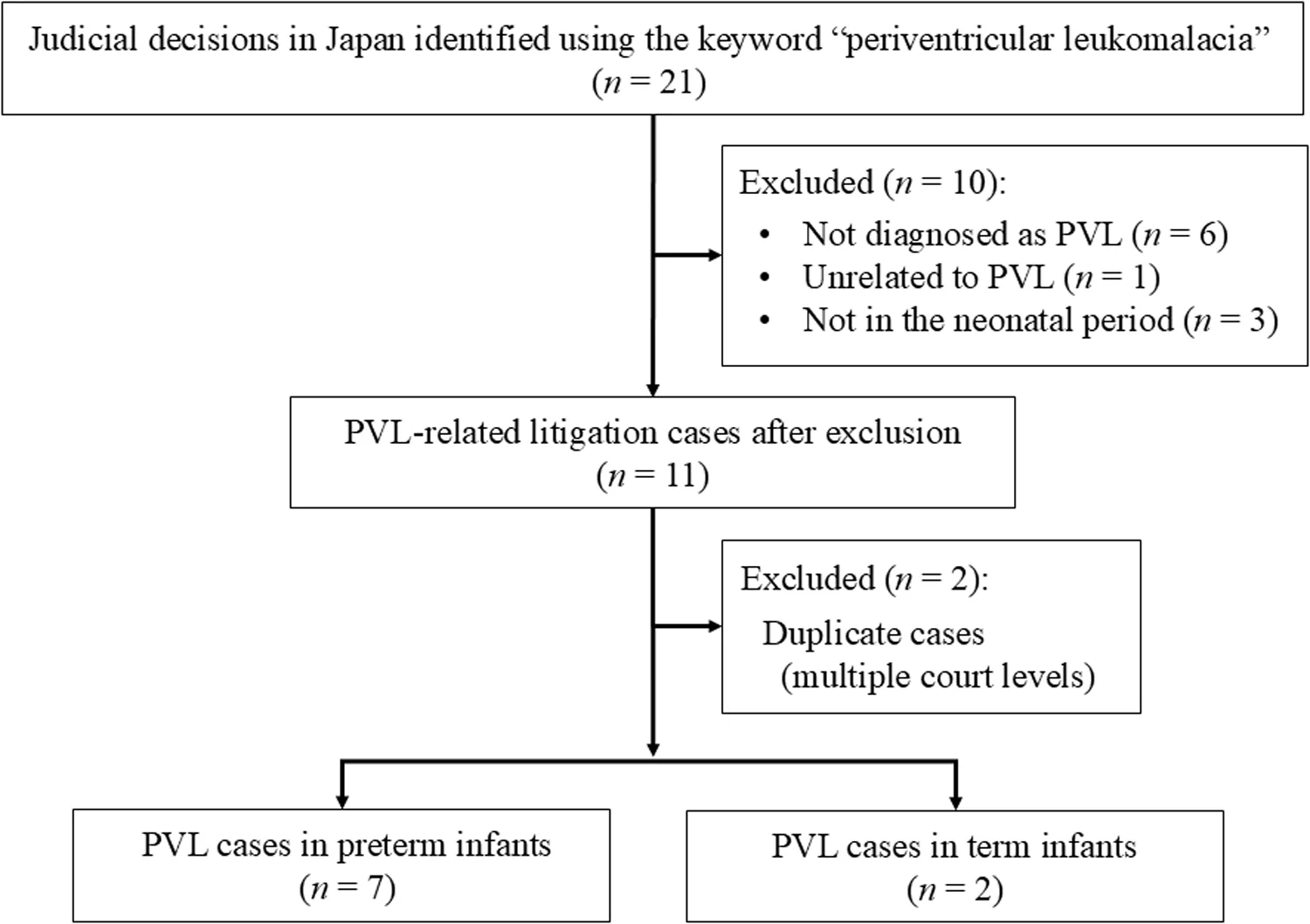
Selection process of periventricular leukomalacia–related litigation cases from a national legal database in Japan. Flow diagram illustrating the identification and selection of periventricular leukomalacia-related medical litigation cases.

A flowchart of case identification and selection is illustrated in Figure 1. A total of 21 judicial decisions were initially identified using the keyword “periventricular leukomalacia.” After excluding cases that were not diagnosed with PVL (*n* = 6), were unrelated to PVL (*n* = 1), or did not occur during the neonatal period (*n* = 3), eleven cases remained. Among these, two duplicate cases arising from multiple court levels were excluded, resulting in nine unique PVL-related cases. Two infants born at term were excluded from further analysis. Seven cases were included in the final analysis.

The clinical characteristics of the seven patients are summarized in Table 1. Gestational age ranged from 27 to 34 weeks. Although the clinical courses and perinatal complications varied considerably among cases, all infants developed CP. The cases were filed between 1991 and 2012, and judgments were issued between 1995 and 2016. Of the seven cases, five resulted in judgments in favor of the plaintiffs at either the trial or appellate level; however, in one case, the appellate court reversed the dismissal by the trial court. The remaining two cases were decided in favor of the defendants.

**Table 1:** Clinical characteristics of seven cases of periventricular leukomalacia-related medical litigation in preterm infants in Japan.

Case	Birth year	Maternal complications	Mode of delivery	Gestational age (weeks)	Birth weight (g)	1-min Apgar score	Neonatal complications	Neurological outcome	Maternal special notes	Neonatal clinical notes
1	1986	Threatened preterm labor	Vaginal delivery (birth in ambulance)	29 + 1	1,060	ND	Respiratory distress syndrome; apnea	Cerebral palsy	Maternal transfer	Mechanical ventilation
2	1991	Threatened preterm labor; premature rupture of the membrane	Vaginal delivery	34 + 4	ND	1	Neonatal asphyxia; hypoxic encephalopathy; Intraventricular hemorrhage	Cerebral palsy; death at a later date	Prolongation of pregnancy after the delivery of the first twin	Mechanical ventilation
3	1992	Placenta previa; threatened preterm labor	Cesarean delivery	31 + 4	1,716	7	Respiratory distress syndrome; pneumothorax	Cerebral palsy	–	Mechanical ventilation; surfactant replacement therapy
4	1993	Threatened preterm labor; fetal distress	Cesarean delivery	33 + 6	2,250	6	Respiratory distress syndrome	Cerebral palsy	–	Mechanical ventilation
5	1998	Threatened preterm labor; maternal hypovolemic shock	Vaginal delivery	28	820	ND	Anemia; apnea	Cerebral palsy	Maternal transfer	Mechanical ventilation
6	2004	Threatened preterm labor	Cesarean delivery	27 + 1	1,142	ND	Apnea	Cerebral palsy	–	Mechanical ventilation
7	2003	Intrauterine death of one fetus in triplet pregnancy	Cesarean delivery	30 + 6	1,168	ND	ND	Cerebral palsy	–	–

This table summarizes the clinical characteristics of seven reported cases of medical litigation related to periventricular leukomalacia in preterm infants in Japan. Data were extracted from published court decisions and related medical records when available. Maternal complications included obstetric conditions before delivery. Neonatal complications refer to major clinical events that occur during the neonatal period. The neurological outcomes were uniformly reported as cerebral palsy in all cases.

ND indicates that the information was not described in the original source. “Maternal transfer” refers to interfacility transfer before delivery.

In addition to neonatal clinical information, information on maternal, obstetric, fetal, and placental factors was reviewed whenever described in the judicial decisions. Information on pregnancy-related maternal complications was available in several cases and is summarized in Table 1. In contrast, information regarding family history, non-obstetric maternal comorbidities, fetal anomalies, and placental pathology was unavailable in most cases because these details are not consistently documented in judicial records. Accordingly, maternal, placental, and fetal factors were reviewed descriptively but could not be evaluated systematically across cases.

### 3.1 Case 1: delayed maternal transfer and inadequate monitoring

A woman hospitalized at 29 weeks of gestation for threatened preterm labor initially improved following tocolytic therapy; nonetheless, uterine contractions subsequently intensified, and transfer to a higher-level facility was arranged. The infant was delivered during ambulance transport, but suffered perinatal asphyxia and later developed severe CP.

The court found a breach of duty of care, citing inadequate monitoring to assess labor progression, delayed recognition of labor advancement, and delayed decision-making regarding maternal transfer. However, with respect to causation, the court emphasized that PVL was closely associated with the intrinsic risks of prematurity and recognized only a limited causal relationship between medical management and neurological outcomes. Consequently, the court awarded substantially reduced damages, restricting compensation to a limited scope.

### 3.2 Case 2: delay in decision-making for rapid delivery in the second twin

In a twin pregnancy at 32 weeks of gestation, the first twin was delivered vaginally. Nevertheless, during the second delivery, persistent variable decelerations associated with malrotation were observed. The repeated application of fundal pressure resulted in asphyxia. The infant subsequently died following the development of hypoxic-ischemic encephalopathy, PVL, and severe CP.

The court determined that an indication for rapid delivery arose when fetal heart rate abnormalities and malrotation persisted with arrested labor progression. They found a breach of duty of care in failing to perform an emergency cesarean delivery, which was considered the only appropriate option. The court further concluded that a timely cesarean delivery could have prevented prolonged fetal hypoxia and severe neurological injury, thereby establishing proximate causation.

### 3.3 Case 3: divergent judgments between trial and appellate courts

An infant born by cesarean delivery at 31 weeks of gestation for placenta previa and threatened preterm labor received surfactant therapy and mechanical ventilation for neonatal respiratory distress syndrome, but later developed CP. The plaintiff alleged inappropriate timing of cesarean delivery and inadequate postnatal respiratory management.

The trial court emphasized physician discretion in obstetric and neonatal management, as well as the multifactorial nature of CP, and denied both breach of duty of care and causation. In contrast, the appellate court identified multiple deficiencies in obstetric and neonatal management, including failure to adequately consider prolongation of pregnancy to promote fetal lung maturation and insufficient postnatal respiratory care. The appellate court constructed a causal chain linking perinatal hypoxia–ischemia to PVL and subsequent CP, and recognized both breach of duty of care and proximate causation.

### 3.4 Case 4: differentiation between periventricular leukomalacia and fetal asphyxia as the cause of cerebral palsy

An infant born at 33 weeks of gestation, requiring respiratory management, was later diagnosed with CP. The central issue was whether the condition was attributable to fetal compromise or PVL caused by premature birth.

At the trial level, the court concluded that CP was more likely caused by PVL due to prematurity than fetal compromise and rejected the plaintiff's claims of obstetric negligence. On appeal, multiple issues were raised, including the etiology of CP, appropriateness of cesarean timing, potential violations of the duty to obtain fetal information, and informed consent. The court emphasized the nonspecific nature of fetal heart rate monitoring findings and the difficulty of diagnosing acute hypoxic events based solely on such data. In addition, the court recognized that continuation of pregnancy in preterm cases was medically reasonable. Ultimately, the claims were dismissed, ruling out breaches of duty or causation.

### 3.5 Case 5: respiratory management and apnea episodes

An extremely low-birth-weight infant born at 28 weeks of gestation developed apnea after extubation and was subsequently diagnosed with PVL and CP. The plaintiff argued that early extubation and inadequate management of apnea contributed to PVL development, whereas the defense emphasized the multifactorial etiology of the condition.

The court held that the pathogenesis of PVL remains incompletely understood and that apnea could not be established as a direct cause. The court also noted that early extubation represented a reasonable clinical strategy for reducing ventilator-associated complications. Accordingly, neither a breach of duty of care nor causation was established.

### 3.6 Case 6: failure to disclose periventricular leukomalacia diagnosis

An extremely low-birth-weight infant born at 27 weeks of gestation demonstrated imaging findings consistent with PVL during the neonatal period; however, the diagnosis was not disclosed to the family. The patient was subsequently diagnosed with CP secondary to PVL at another institution.

This case focused on whether there had been a breach of duty to inform, as well as deficiencies in follow-up and management. The court held that even in the absence of an established effective treatment, disclosure of the diagnosis was essential for family decision-making and, therefore, constituted a breach of the duty to explain. Nevertheless, early diagnosis of CP was difficult, given the prevailing medical standards, and did not constitute a breach of duty of care. No causal relationship was found between non-disclosure and clinical outcomes, and compensation was limited to emotional damage.

### 3.7 Case 7: early delivery in a multiple pregnancy

In a triplet pregnancy, the intrauterine death of one fetus led to emergency cesarean delivery at 30 weeks of gestation. One surviving infant was later diagnosed with PVL and developed severe CP.

The plaintiff argued that the pregnancy should have been prolonged after intrauterine death. The court recognized that the continuation of pregnancy may have reduced the risk of PVL and found a breach in the duty of care in the decision to proceed with an early delivery. The court also established proximate causation between the timing of delivery and the neurological outcomes, awarding substantial damages. This decision was later criticized by professional obstetric and neonatology societies who emphasized the multifactorial nature of PVL and noted that late-onset circulatory collapse (18) had also been discussed as a possible contributing factor, arguing that gestational age alone could not fully explain the outcome. The case was ultimately settled at the appellate stage.

### 3.8 Cross-case analysis

#### 3.8.1 Distribution of clinical issues

The clinical issues identified across the seven cases involved obstetric management, neonatal management, or both, depending on the presumed timing and mechanism of injury. The key issues and outcomes of the seven cases are summarized in Table 2. The main issues in obstetric care included the timing of delivery, adequacy of fetal monitoring, decisions regarding maternal transfer, and appropriateness of interventions during labor, including the indication for rapid delivery. Neonatal care issues primarily involved respiratory management, monitoring practices, and the organization of treatment systems. Informed consent was also identified as an issue in some cases. Issues regarding both obstetric and neonatal management were identified in Case 3.

**Table 2:** Key legal issues and court judgments in seven cases of periventricular leukomalacia-related medical litigation in Japan.

Case	Year Fild	Court Level	Obstetric Care					Neonatal Care			Causation	Outcome
			Treatment System	Monitoring	Maternal Transfer	Timing of Delivery	Informed Consent	Monitoring	Treatment System	Informed Consent		
1	1991	District Court	**N**	**Y**	**Y**	–	–	–	–	–	**P**	Plaintiff
2	1993	District Court	–	–	–	**Y**	**N**	–	–	–	**Y**	Plaintiff
3	1998	District Court	–	–	–	**N**	–	**N**	**N**	–	**N**	Defendant
	2003	Appellate Court	–	–	–	**Y**	–	**Y**	**Y**	–	**Y**	Plaintiff
4	Unknown	District Court	–	**N**	–	**N**	**N**	–	–	–	**N**	Defendant
	2001	Appellate Court	–	**N**	–	**N**	**N**	–	–	–	**N**	Defendant
5	2003	District Court	–	–	–	–	–	**N**	**N**	–	**N**	Defendant
6	2006	District Court	–	–	–	–	–	–	**N**	**Y**	**N**	Plaintiff
7	2012	District Court	–	–	–	**Y**	–	–	–	–	**Y**	Plaintiff

This table summarizes the major legal issues and court judgments in seven medical litigation cases related to periventricular leukomalacia in preterm infants in Japan. The issues analyzed were categorized into obstetric and neonatal care, including treatment systems, monitoring practices, maternal transfer, timing of delivery, and informed consent. Symbols indicate the court's evaluation of each issue as follows: “Y” indicates that the court explicitly recognized breach of duty or causation. “N” indicates explicit denial. “P” indicates partial or limited recognition of causation. “–” indicates that the issue was not substantially addressed in the judicial decision. “Outcome” indicates the final judgment at each court level (plaintiff or defendant). One case (Case 7) subsequently settled at the appellate level.

#### 3.8.2 Recognition of breach of duty

Breaches of duty of care were recognized in several cases. Identified breaches included deficiencies in monitoring systems, delays in clinical decision-making, and inadequate intervention. In obstetric care, breaches of duty were associated with delayed decisions regarding delivery timing and failure to initiate appropriate interventions during labor (Cases 1, 2, 3, and 7). In contrast, breach of duty of care was not consistently recognized, particularly in cases where clinical management was considered consistent with accepted medical practice (Cases 5 and 6). However, in Case 6, a breach of duty to inform was recognized, independent of clinical management.

#### 3.8.3 Evaluation of causation

The evaluation of causation varied across cases. In Cases 2, 3, and 7, the courts recognized causation by linking a sequence of clinical events, such as intrapartum hypoxia, prematurity, and subsequent neurological deterioration, to the final neurological outcome. In contrast, in Cases 4 and 5, the court denied causation because the multifactorial nature of PVL and the uncertainty regarding the timing and mechanism of injury made it difficult to establish a direct relationship between the medical management and neurological outcomes. In Case 1, a breach of duty was recognized; however, the causal relationship between medical management and neurological outcomes was acknowledged only to a limited extent.

#### 3.8.4 Variability in judicial decisions

Judicial decisions varied across cases. Differences were observed in recognition of a breach of duty, evaluation of causation, and final outcomes. In addition, in Case 3, the judgment differed between the trial and appellate courts: the trial court denied both breach of duty and causation, whereas the appellate court recognized both based on the same case background. These findings indicate the variability in judicial evaluations across cases and between courts.

## 4 Discussion

The key legal characteristics identified across the seven PVL-related litigation cases are presented in Table 3. To interpret these findings, it is important to consider the institutional context of medical malpractice litigation in Japan. Unlike jurisdictions such as the United States, where jury trials, extensive pre-trial discovery, and party-retained expert witnesses may play major roles in malpractice litigation, medical malpractice cases in Japan are generally adjudicated by professional judges in ordinary civil proceedings (19, 20). Medical evidence is evaluated primarily through documentary records, arguments presented by both parties, and expert opinions when obtained by the court or submitted by the parties. Since the late 1990s and early 2000s, when medical accidents became a major social concern in Japan, several reforms have been introduced to improve medical safety and the management of medical litigation, including specialized handling of medical cases by major district courts and the development of non-litigation mechanisms such as medical alternative dispute resolution, the Medical Accident Investigation System, and the Japan Obstetric Compensation System for Cerebral Palsy (20, 21). Therefore, the judicial decisions analyzed in this study should be understood within a medico-legal system that combines civil liability assessment with broader institutional efforts toward patient safety, dispute resolution, and prevention of recurrence. The following sections discuss these findings in relation to the structure of perinatal care, evaluation of causation, and broader implications for clinical practice.

**Table 3:** Summary of legal characteristics of periventricular leukomalacia-related medical litigation cases in Japan.

Category	Number of cases	Interpretation
Total cases	7	–
Cases involving obstetric management issues	5	Obstetric decision-making, particularly timing of delivery, was frequently evaluated
Cases involving neonatal management issues	3	Postnatal care played a central role in legal evaluation
Cases involving both obstetric and neonatal factors	1	Supports the continuum of perinatal care across pre- and postnatal periods
Cases in which causation was recognized	4	Confirms the central role of causation in determining legal responsibility
Cases in which legal liability was recognized	5	Closely associated with the establishment of causation
Cases with discrepancy between court levels	1	Indicates variability in judicial interpretation
Cases in which prematurity was explicitly cited in legal reasoning	4	Reflects the dual role of prematurity as both a limiting and aggravating factor
Cases involving system-level factors	6	Highlights the importance of healthcare system factors in legal judgments
Cases with neonatal care negligence recognized	1	Emphasizes the significance of postnatal management
Cases with explanation duty violation	1	Suggests that informed consent can serve as an independent basis for liability
Cases with substantial compensation awards (> 100 million yen)	1	Severe outcomes may be associated with substantial compensation, although numbers are limited

“Obstetric management issues” include maternal transfer and delivery timing. “Neonatal management issues” include monitoring, respiratory management, and postnatal care practices. “System-level factors” include monitoring systems, decision-making processes, and timing of clinical interventions. The compensation amounts were based on publicly available court records.

### 4.1 Periventricular leukomalacia-related litigation reflects the continuum of perinatal care in the context of clinical uncertainty

The analysis demonstrated that PVL-related litigation may involve obstetric management, neonatal management, or both, depending on the presumed timing and mechanism of injury. This distribution of disputed issues across different phases of perinatal care reflects the uncertainty surrounding the timing of injury and mechanisms underlying PVL. As PVL is a multifactorial disorder and the precise timing of brain injury cannot always be determined, legal responsibility may be attributed to obstetric management, neonatal management, or both, making medico-legal evaluation particularly challenging (10, 11). Accordingly, legal responsibility was assessed not in relation to a single time point or isolated clinical act, but across multiple phases of clinical decision-making and interventions spanning the antenatal, intrapartum, and postnatal periods. Often, it can be difficult to attribute responsibility to a single care phase. Consequently, legal evaluations tend to encompass both obstetric and neonatal management. This was most clearly demonstrated in Case 3, in which both obstetric and neonatal management were evaluated across different phases of care. In the appellate decision, causation was established on the basis that obstetric management contributed to a clinical context in which prematurity became a critical factor. Therefore, subsequent deficiencies in neonatal respiratory care were interrelated and collectively influenced the outcome. This case illustrates that obstetric and neonatal management may be evaluated not as independent domains but as an integrated process of perinatal care. Moreover, this decision underscores not only the interrelationship of obstetric and neonatal management but also the importance of timely decision-making during labor, highlighting the need for close collaboration between obstetricians and neonatologists within integrated perinatal care systems.

### 4.2 Causation as the central determinant in judicial evaluation

A key finding of this study was the central role of causation in determining legal liability. In clinical practice, the multifactorial nature of PVL can make it difficult to attribute its outcomes to a single cause (10). In addition, uncertainties in developmental prognosis (22) and limited evidence regarding the effectiveness of early interventions (23, 24) can further complicate clinical interpretation. However, unlike clinical medicine, judicial decision-making requires the construction of a coherent causal link between medical conduct and outcomes despite substantial uncertainty surrounding PVL. Consequently, even when a breach of duty is recognized, the acceptance of claims tends to be limited if causation was not sufficiently established. In Japanese medical litigation, the difficulty of proving causation in multifactorial diseases often leads to denial of liability (25, 26). In Case 1, the claims were only partially accepted despite the recognition of a breach of duty; a proximate causal relationship between the medical act and PVL was not fully established. This case demonstrates that the recognition of negligence alone is legally insufficient for liability without a clear causal linkage. In contrast, when clear clinical events, specifically intrapartum hypoxia, were documented, courts were more likely to affirm causation by constructing a continuous temporal sequence linking these events to neurological outcomes. In Case 2, persistent fetal heart rate abnormalities and prolonged labor supported the conclusion that timely cesarean delivery could have prevented adverse outcomes. Similarly, in the appellate decision in Case 3, a sequence involving postnatal respiratory failure, hypoxia, reduced cerebral perfusion, PVL, and CP was recognized as a plausible causal chain. Case 7 likewise demonstrated that judicial recognition of causation may be based on a coherent temporal sequence despite persistent uncertainty regarding the timing and mechanisms of PVL. It should also be acknowledged that currently available methods of fetal surveillance have inherent limitations in the prospective identification of fetuses at risk of subsequent brain injury. Consequently, obstetric decisions are often prospectively required on the basis of incomplete clinical information rather than definitive evidence of evolving fetal brain injury.

These findings indicate that although the multifactorial nature of PVL is recognized, judicial determinations of causation are strongly influenced by the clarity of the clinical course and perceived avoidability of the outcome. Furthermore, they highlighted the structural difference between medicine and law: whereas clinical medicine explicitly acknowledges uncertainty regarding the pathogenesis and timing of PVL, the judiciary requires a degree of logical coherence to attribute responsibility. Consequently, judicial assessment of causation inevitably involves interpretation despite persistent uncertainty regarding the pathogenesis of PVL.

### 4.3 Context-dependent interpretation of prematurity in judicial reasoning

Prematurity was interpreted differently across cases, and its legal significance varied depending on the context. In Cases 1, 4, and 5, prematurity was regarded as an inherent, unavoidable biological risk (27) and thus served as a key factor supporting the denial of causation. In these cases, the courts emphasized the difficulty in distinguishing the effects of medical management from the underlying vulnerability associated with prematurity. Nevertheless, in Case 3, prematurity was interpreted differently between judicial levels, whereas the trial court treated it as a liability-limiting factor, the appellate court regarded it as a condition requiring heightened clinical vigilance, thereby reinforcing the duty of care. Furthermore, the relative importance of prematurity was reduced in cases involving clearly identifiable acute events. For example, in Case 2, management-related factors were given greater weight. Importantly, the litigation in these cases concerned the indication for and timing of cesarean delivery rather than cesarean delivery itself as an independent etiological risk factor for PVL. These findings suggest that prematurity does not have a fixed legal meaning but can be interpreted flexibly, depending on the clinical context and the judicial construction of causation.

### 4.4 Dual role of system-level and decision-making factors in liability assessment

The present study demonstrates that courts evaluate system-level factors as well as individual acts. Liability was assessed in relation to monitoring systems, decision-making processes, the timing of interventions, and communication practices. In Case 1, deficiencies in monitoring and delays in maternal transfer were central to the recognition of breach of duty, indicating the importance of system capacity in perinatal management (28, 29). In Case 6, the failure to disclose the diagnosis highlighted the role of communication systems in legal evaluations. Conversely, in Case 2, the appropriateness of a specific clinical decision, namely the timing of rapid delivery, was directly linked to liability. Thus, liability in PVL litigation appears to have a two-layered structure consisting of (A) system-level factors, such as monitoring and transport systems (30), and (B) individual decision-making factors, including the timing and method of intervention. These findings indicate that liability in PVL-related litigation is structured by both system-level factors and individual clinical decisions, suggesting a dual framework in which organizational conditions (29) and the quality of clinical decision-making are jointly evaluated. Such evaluations are particularly important in the management of PVL, where clinical decision-making is frequently required under conditions of substantial uncertainty regarding the timing and mechanisms of brain injury.

### 4.5 Divergence between medical understanding and judicial construction of causation

This study suggests a discrepancy between medical knowledge and judicial reasoning. Although clinical medicine recognizes the multifactorial and uncertain nature of PVL, judicial decisions often construct causation based on temporal relationships and logical coherence. When acute intrapartum or perinatal events are clearly documented, disorders that are medically understood as multifactorial may sometimes be interpreted as a single causal sequence. This tendency was evident in the appellate decision for Case 3, in which causation was affirmed through a sequence of clinical progression linking perinatal hypoxia–ischemia, respiratory insufficiency, PVL, and subsequent CP. In contrast, the interpretation for Case 7 was more controversial, with the prolongation of gestation after intrauterine fetal death in a triplet pregnancy being linked by the court to the prevention of PVL despite substantial medical uncertainty regarding the timing and mechanism of injury. This interpretation was subsequently questioned by professional obstetrics and neonatology societies. These findings reflect fundamental differences in the evaluative frameworks of medicine and law, particularly in the construction of causation. In certain cases, the judicial interpretation of causation may exceed the degree of certainty generally accepted in clinical medicine. This discrepancy may become particularly pronounced in multifactorial neonatal conditions such as PVL, where the timing and mechanism of injury often remain uncertain.

The present findings also illustrate the broader challenge of decision-making under conditions of clinical uncertainty. In the evaluation of PVL, diagnostic evaluation and prognostic assessment frequently rely on incomplete information because both the timing and mechanisms of brain injury may remain uncertain. Scher emphasized that uncertainty is an inherent characteristic of fetal-neonatal neurology and that clinical decision-making should integrate evolving evidence through transdisciplinary collaboration and shared decision-making (31, 32). Likewise, recent discussions on causal inference in medical research have highlighted that causal interpretation should explicitly acknowledge underlying assumptions and the limitations of available evidence, particularly when conclusions are derived from observational data (33). Although judicial decision-making necessarily differs from clinical reasoning, both disciplines ultimately confront the common challenge of reaching decisions despite incomplete certainty. The present findings suggest that medico-legal evaluation of PVL may benefit from greater recognition of the inherent uncertainty surrounding its pathogenesis and timing of injury. More broadly, these observations are consistent with well-recognized cognitive challenges in human judgment under conditions of uncertainty, including heuristic reasoning and hindsight bias, as described in decision science (34).

### 4.6 Independent role of the duty to inform in legal responsibility

Although not always a primary issue, the duty to inform has emerged as an independent basis for liability. In Case 6, a breach of duty to inform was recognized despite the absence of a causal relationship with the clinical outcome. This case indicates that legal responsibility in PVL-related litigation may arise solely from poor communication. This issue is further complicated by the substantial prognostic uncertainty associated with PVL, which makes decisions regarding the timing and manner of disclosure particularly challenging. Although PVL is a well-recognized risk factor for CP, not all affected infants develop neurological impairments (35), and many remain asymptomatic for several months before clinical manifestations become evident (13). In addition, the disclosure of a PVL diagnosis may impose substantial psychological distress on families (36). Therefore, clinicians must carefully consider multiple factors, including parents' psychological condition, their understanding of disability, their capacity to process medical information, and their level of trust in the physician–family relationship when determining how and when to provide such information. In Case 6, the court acknowledged that the timing of the disclosure should be left to the physician's discretion. This finding adds to the complexity of medico-legal evaluation under conditions of persistent prognostic uncertainty and highlights the importance of individualized communication in perinatal care.

### 4.7 Clinical implications for decision-making and risk management in perinatal care

Our findings have several clinical implications. First, the systematic documentation of clinical decision-making processes is essential, particularly in situations involving uncertainty and rapidly changing clinical conditions. Second, system-based care frameworks, including structured monitoring systems and shared decision-making processes, are important in terms of both clinical quality and legal defensibility (37). Third, clinicians must explicitly communicate with families regarding the uncertainty and limitations of prognostic prediction in multifactorial conditions, such as PVL (38). Finally, obstetric and neonatal care should be managed as a continuous and integrated process, rather than as separate specialties. Taken together, these findings underscore that, under conditions of clinical uncertainty, careful documentation of clinical reasoning together with multidisciplinary collaboration is essential not only for optimal patient care but also for medico-legal accountability.

### 4.8 Limitations

This study had some limitations. First, the number of analyzed cases was limited because PVL-related medical litigation is relatively rare in Japan. Second, only judicial decisions that resulted in publicly available court judgments were eligible for inclusion, which potentially limits the generalizability of the findings. In Japan, many medical disputes are resolved through settlement or alternative dispute resolution without a final published judgment (19). Consequently, the cases analyzed in this study represent only a subset of all PVL-related medico-legal disputes and may preferentially be representative of unresolved legal issues or contested judicial reasoning. Third, this study relied on judicial decisions, which are reconstructed legal narratives rather than original medical records and therefore may not fully capture the complexities of clinical practice. Although information on maternal, fetal, and placental factors was reviewed whenever described in the judicial decisions, such information was often incomplete or unavailable, particularly regarding family history, maternal non-obstetric comorbidities, fetal anomalies, and placental pathology. Consequently, the present analysis could not comprehensively evaluate maternal, placental, or fetal factors beyond the information explicitly documented in the judicial records. The inherent uncertainty surrounding the pathogenesis and timing of PVL should also be considered when interpreting the legal reasoning described in the present study. Nevertheless, because all publicly available judgments meeting the predefined eligibility criteria were included through a nationwide search without date restrictions, the present study provides a reasonably comprehensive qualitative analysis of judicial reasoning in reported PVL-related litigation in Japan.

### 4.9 Future directions and concluding remarks

Future research should include a broader range of cases, including those resolved through settlement, and incorporate international comparative analysis. Developing a theoretical framework for integrating medical uncertainty into judicial decision-making is an important challenge. In particular, evaluating the relationship between intrapartum decision-making and outcomes remains a key issue. Furthermore, the relationship between judicial trends and the Japanese Obstetric Compensation System is critical to consider. Introduced in 2009 (21), this system initially excluded cases of CP in infants born before 32 weeks of gestation, as the condition was primarily attributable to preterm birth. However, with advances in perinatal care and a decline in the incidence of CP among preterm infants (39), the eligibility criteria were expanded in 2022 to include infants born at ≥ 28 weeks' gestation. This institutional change suggests a shift away from viewing prematurity as an unavoidable factor. Although preterm birth remains a significant risk factor, it may increasingly be viewed as a condition modifiable by clinical management. This perspective aligns with the present findings, which demonstrate that preterm birth may be interpreted either as an unavoidable factor or as one that heightens the duty of care. Overall, an integrated understanding of the interactions between clinical practice, judicial reasoning, and institutional frameworks represents an important direction for future research. Another promising avenue for future research is examining how prospective clinical decision-making under conditions of uncertainty is subsequently evaluated through retrospective medico-legal review, including the potential influence of hindsight bias on judicial reasoning. Such approaches may contribute to improving both clinical practice and medico-legal risk management.

In conclusion, PVL-related litigation involves medico-legal evaluation across multiple phases of perinatal care because responsibility may be attributed to different stages of management under conditions of clinical uncertainty. Rather than being confined to a single medical act, legal responsibility is assessed in relation to the timing of injury, the sequence of clinical events, and the overall context of clinical decision-making. While causation remains the central determinant of liability, courts may construct causal relationships even in the context of multifactorial uncertainty. These findings suggest that both system-level factors and individual clinical decision-making are critical in the legal evaluation of care. An integrated understanding of clinical practice, judicial reasoning, and institutional frameworks is essential for improving perinatal care, supporting clinical decision-making, and strengthening medico-legal risk management.

## Data Availability

The data analyzed in this study is subject to the following licenses/restrictions: The judicial decisions analyzed in this study were obtained from D1-Law.com (Daiichi Hoki, Tokyo, Japan) and related published legal sources. Access to these materials is restricted by subscription and copyright regulations because the original judicial documents are not owned by the author and are subject to licensing restrictions, they cannot be made publicly available. All information relevant to the findings of this study is presented within the article. Information regarding access to these materials is available at https://www.d1-law.com/. Access is subject to subscription and licensing restrictions.
